# The involvement of small heat shock protein in chemoresistance in ovarian cancer - *in vitro* study

**DOI:** 10.17179/excli2021-3706

**Published:** 2021-05-25

**Authors:** Aleksandra Wyciszkiewicz, Michal S. Lach, Joanna P. Wróblewska, Marcin Michalak, Wiktoria M. Suchorska, Alicja Kalinowska, Slawomir Michalak

**Affiliations:** 1 Department of Neurology, Division of Neurochemistry and Neuropathology, Poznan University of Medical Sciences, Przybyszewskiego 49, 60-355 Poznan, Poland; 2 Radiobiology Laboratory, Greater Poland Cancer Centre, Garbary 15, 61-866 Poznań, Poland; 3 Department of Electroradiology, Poznan University of Medical Sciences, Garbary 15, 61-866 Poznań, Poland; 4 Department of Tumor Pathology and Prophylaxis, Poznań University of Medical Sciences, Garbary 15, 61-866 Poznań, Poland; 5 Department of Oncologic Pathology, Greater Poland Cancer Centre, Garbary 15, 61-866 Poznań, Poland; 6 Surgical, Oncological, and Endoscopic Gynaecology Department, Greater Poland Cancer Centre, Garbary 15, 61-866 Poznan, Poland

**Keywords:** ovarian cancer, chemoresistance, small HSPs, exosomes

## Abstract

Ovarian cancer is the most deadly gynecologic malignancy worldwide. Although the primary response to chemotherapy is high, the majority of patients will develop resistance against applied treatment. In this study, we focused on resistance to cisplatin, a first-line drug used for the treatment of ovarian cancer. The mechanism of the resistance development process is widely described, but there is a lack of information about the involvement of members of small heat shock proteins (HSPs) and their transport via exosomes. In this study, we used two cell lines: A2780 and SKOV3, and their cisplatin-resistance variants: A2780 CDDP and SKOV3 CDDP. We have shown that the expression of three small HSPs (HSPB5, HSPB6, and HSPB8) in cisplatin-resistant cell lines differs from their sensitive counterparts. Further, we isolated exosomes and determined the small HSPs in their cargo. In A2780 WT we observed a low amount of HSPB5 and HSPB6. We did not observe the expression of small HSPs in the SKOV3 cell line in both sensitive and resistant variants. Our data suggest the involvement of small HSPs in drug resistance of ovarian cancer and their presence is not related to exosomal transport. Analysis of the biological consequences of the imbalance of small HSPs expression in cisplatin resistance needs further investigation.

## Introduction

Ovarian cancer (OvCa) is still one of the most challenging cancers in terms of improvement of existing therapies with the highest mortality rate among gynecologic malignancies. A few causes are responsible for the occurrence of those circumstances. Firstly, lack of effective screening based on biomarkers or on ultrasound examination and asymptomatic early stages of the disease, lead to its manifestation at advanced stages with a significantly reduced 5-year survival rate (Momenimovahed et al., 2019[38]; Siegel et al., 2016[51]). Secondly, the frequently occurring development of chemoresistance is the main cause of disease recurrence. The standard approach for OvCa treatment is based on surgery followed by adjuvant platinum- or taxane-based chemotherapy (Cornelison et al., 2017[15]). Most of the patients show a good initial response to applied treatment, but among them, over two-thirds will develop a resistant phenotype of the disease (Cooke and Brenton, 2011[14]). 

Two types of chemoresistance can be distinguished. First one, intrinsic, where the cancer cells are inherently resistant to applied treatment due to the natural occurrence of mechanisms that are responsible for decreased uptake of drugs or inhibition of apoptosis. The other is acquired, which can be induced during applied treatment, through adaptation and development of protective mechanisms of initially sensitive cells to the cytotoxic agents (Rubin et al., 1999[49]; Amstrong, 2002[2]; Christie and Bowtell, 2017[11]; Cornelison et al., 2017[15]). In both processes, several macromolecules were identified and marked as potential biomarkers of therapeutic response for advanced ovarian carcinoma (Osterberg et al., 2009[43]; Têtu et al., 2008[58].; Pokhriyal et al., 2019[45]; Cornelison et al., 2017[15]). There is a lack of one and consistent biomarker due to complex biology and distinct origin of different types of ovarian cancer (Kurman and Shih, 2010[29]; Matulonis et al., 2016[36]). 

One of the additional mechanisms responsible for relapse and failure of applied treatment is the involvement of secreted specialized exosomes. These double-membrane structures with the viral-like size (range 30-150 nm) are now intensively studied, due to their huge potential as a specific biomarker of several tumors and response to applied treatment (Szajnik et al., 2016[56]; Kalluri and LeBleu, 2020[23]). They contain a wide range of biologically active molecules. Moreover, the composition of their cargo depends on the cellular physiological state and exposure of cells to chemical or physical factors (Suchorska and Lach, 2016[53]; Anand et al., 2019[1]). 

One of the proteins involved in cancer progression and resistance to applied treatment *via* exosomal transport are chaperones, also known as heat shock proteins (HSPs) (Rappa et al., 2012[47]; Ciocca and Calderwood, 2005[12]). Based on the molecular size of HSPs, the two main groups of HSPs could be distinguished as a large - HSP110, HSP90, HSP70, HSP60, HSP40 - and the small one HSPB1/HSP27, HSP5/alpha-B Crystallin, HSPB6/HSP20, and HSPB8/HSP22, which are mostly ubiquitously expressed while HSPB2 and HSPB7 are essentially restricted to heart and muscles, HSPB4/alpha-A Crystallin is lens-specific and HSPB9 and HSPB10 are both testis-specific (Rappa et al., 2012[47]; Treweek et al., 2015[59]; Kampinga et al., 2009[24]). Recent studies regarding large HSPs, such as HSP70, HSP90, and HSP60 demonstrated that those proteins localized on the surface of exosomes, secreted by normal and tumor cells, could be key players in intercellular cross-talk (Campanella et al., 2014[7]; Lancaster and Febbraio, 2005[30]). Moreover, they interact with survivin, a member of the inhibitor of apoptosis (IAP) protein family, which in consequence increase the survival of cancer cells to applied therapy (Khan et al., 2011[25]; Gonda et al., 2018[19]).

In terms of chemoresistance, the involvement of small HSPs and their secretion through the exosomal pathway in ovarian cancer is not a well-known phenomenon. They are identified as pro-cancer drivers and can be involved in the activation of anti-apoptotic response, cancer cell proliferation, and metastasis development (Cohen et al., 2010[13]; Hoter and Naim, 2019[21]; Sun and MacRae, 2005[54]). The most well-known member among small HSPs associated with poor prognosis in ovarian cancer is HSPB1/Hsp27 (Langdon et al., 1995[31]; Olejek et al., 2009[42]). Another small Hsp identified in the ovarian cancer cell line is HSPB8/Hsp22, which expression was correlated with TGFα induced migration of ovarian cancer cells (Suzuki et al., 2015[55]). In our previous study, we identified the presence of three small HSPs (Alpha-B Crystallin/HSPB5, Hsp20/HSPB6, Hsp22/ HSPB8) in serum, peritoneal fluid, and in isolated exosomes in patients with ovarian cancer which was positively correlated with markers of the cytotoxic immune response (Wyciszkiewicz et al., 2019[62]). 

Given the lack of data about the role of small HSPs (Hsp20, Hsp22, and alpha-B Crystallin) in chemoresistance in ovarian cancer, we aimed to identify and compare the expression of Hsp20 (HSPB6), Hsp22 (HSPB8), and alpha-B Crystallin (HSPB5) in two cell lines: A2780 and SKOV3, both in two variants: wild type and cisplatin-resistant. We also aimed to analyze and point out the differences between the Hsp expression profile between exosomes derived from wild-type, and cisplatin-resistant cell lines.

## Material and Methods

### Cell culture 

Two ovarian cancer cell lines were used in this study: A2780 and SKOV3 (ATCC, Manassas, VA, USA). From each of these cell lines were generated cisplatin-resistant cell variants named as follows: A2780 CDDP and SKOV3 CDDP. The cell culture conditions and derivation of cisplatin-resistant cell lines were conducted and confirmed as previously described (Michalak et al., 2020[37]).

### Isolation of exosomes 

The exosomes were isolated using serial differential centrifugations. Briefly, cells were washed twice with Dulbecco's phosphate-buffered saline (DPBS, Biowest, Nuaillé, France) and a serum-free medium was added. After 48 hours the conditioned medium was collected and centrifuged for 10 minutes at 800 x g and then for 20 minutes at 3000 x g at 4 °C to eliminate larger vesicles and cellular debris. The supernatant was collected and stored at -80 °C until further use. After thawing, approximately 500 ml conditioned medium was filtered through a 0.2 μm membrane and concentrated using 100 kDa cut-off centrifugal filters (Merck KGa, Darmstadt, Germany). The concentrated conditioned medium was diluted 1:1 with DPBS and centrifuged at 120,000 x g for 90 minutes at 4 °C (Ti 70.1 rotor, ultracentrifuge Beckman coulter L7-65 both provided by Beckman Coulter, Munich, Germany). The obtained exosomal pellet was resuspended in DPBS or RIPA buffer according to further analysis. The concentration of exosomes was determined using Pierce™ BCA Protein Assay Kit (Thermofisher, San Jose, CA, USA) according to the manufacturer's instructions.

### Acetylcholinesterase (AChE) activity assay 

To confirm the presence of exosomal acetylcholinesterase activity in the isolated exosomes sample, 5 μg of exosomal protein was diluted in 50 μl of DPBS and mixed with 50 μl of freshly prepared reaction mixture consisted form 0.2 mM 5,5'-dithiobis(2-nitrobenzoic acid) and 2.5 mM acetylthiocholine (both obtained from Sigma-Aldrich, St. Louis, MO, USA) and transferred to 96-well plate. After 30 minutes of incubation at 37 °C, the absorbance was measured at 405 nm using a plate reader (Multiskan FC, Thermofisher, San Jose CA, USA). As a control, DPBS mixed with reaction mixture was used.

### RT-qPCR analysis 

RNA was isolated using Direct-zol RNA MiniPrep (Zymoresearch, Irvine, CA, USA) followed by suspension of 1x10^6^ in TRI reagent (Sigma-Aldrich, St. Louis, MO, USA). Next, 1 µg of total RNA was reverse transcribed using the iScript kit (Bio-Rad, Hercules, CA, USA) according to the manufacturer's protocol. The cDNA was amplified in a total volume of 20 µl and diluted 20 times. Further, the analysis of expression of genes - *HSPB5* (Forward: 5'-GAGGTGCATGGAAAACATGA-3'; Reverse: 5'-GATGAAGTAATGGTGAGAGGGTCT-3'; Probe no. 33), *HSPB6* (Forward: 5'-CACCCTCGCTCTCACACC-3'; Reverse: 5'-AGTGCTGGTAGGGTCTGGAA-3'; Probe no. 78), *HSPB8* (Forward: 5'-CCAGGTCCCTCCTTACTCAA-3'; Reverse: 5'-CCAGGTCCCTCCTTACTCAA-3'; Probe no. 60) - was analyzed using RT-qPCR. As a reference gene for determination relative expression Glyceraldehyde 3-phosphate dehydrogenase (*GAPDH*) gene was used (Universal ProbeLibrary Human GAPDH Gene Assay, Roche Molecular Systems, Inc, Basel, Switzerland). The PCR reaction was carried out in CFX96 Touch Real-Time Detection System (Bio-Rad Hercules, CA, USA) in total 10 µl volume, which consisted of 2.5 µl cDNA, FastStart Essential DNA Probes Mix, and specific probes (both provided by Roche Molecular Systems, Inc, Basel, Switzerland). 

### Western blot 

Total proteins were extracted from cell lines and exosomes using RIPA lysis buffer mixed with proteases inhibitor cocktail (both provided by Sigma Aldrich, St. Louis, MO, USA). Protein concentrations were determined using the Pierce™ BCA Protein Assay Kit kit (Thermofisher, San Jose, CA, USA). The procedure was performed like previously described (Wyciszkiewicz et al., 2019[62]). Briefly, 10 μg of the sample was mixed with reducing Laemmli-buffer and was loaded on 4-20 % Tris-glycine sodium dodecyl sulfate-polyacrylamide gels (Bio-Rad, Hercules, CA, US), and electrophoresed. Proteins were transferred onto polyvinylidene difluoride membrane (Bio-Rad, Hercules, CA, US). After blocking with 5 % non-fat dry milk (Bio-Rad, Hercules, CA, US) in Tris-buffered saline supplemented with 0.05 % Tween-20 (TBST) for 2 h at room temperature, blots were incubated overnight at 4 °C with appropriate primary antibodies as listed in Table 1(Tab. 1). The next day, membranes were washed three times in TBST and incubated with secondary antibodies conjugated with HRP for 2 h at room temperature and washed two times in TBST and then in TBS. The signal of the protein of interest was detected using Clarity Western ECL Substrate (Bio-Rad, Hercules, CA, US) and documented with Chemidoc Touch System (Bio-Rad, Hercules, CA, US). To normalize protein expression, we used 0.01 % Ponceau S staining. The intensity was measured using Image Lab Software (ver. 6.0.1, Bio-Rad Laboratories Ltd., CA, USA).

### Statistical analysis

The statistical analyses of gene expression and Western blot analysis (Welch's t-test) were performed using the GraphPad Prism 6 package (Graph Pad Software, San Diego, CA, USA). The data were deemed significant at p < 0.05.

## Results

### The chemoresistance induces adverse expression of small HSPs

**Figure 1:**
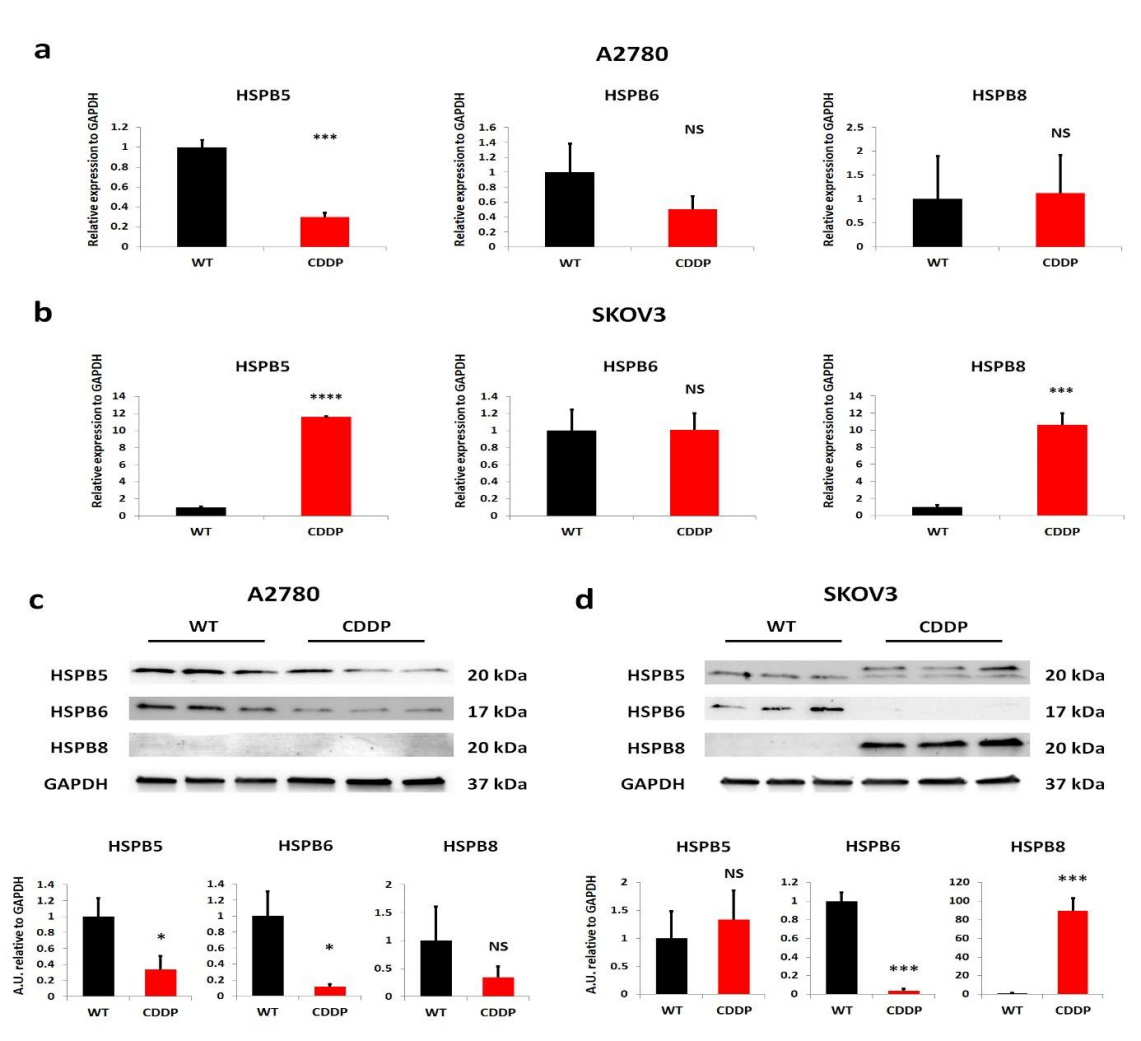
The expression of small HSPs in cisplatin-resistant OvCa cell lines differs from their sensitive counterparts. (a, b) RT-qPCR analysis of mRNA levels of *HSPB5*,* HSPB6*, and *HSPB8* in both A2780 and SKOV3 cell lines, and their sensitive and resistant variants. Relative expression levels of genes were normalized using GAPDH. The bars represent a mean ± SD expression of transcript (n=3). *: *p* < 0.05; **: *p* < 0.01; ***: *p* < 0.001; **** - p by Welch's *t*-test. (c, d) Western blot evaluation of expression of HSPB5, HSPB6 and HSPB8 in whole cell lysates derived from A2780 and SKOV3 cell lines and their sensitive and resistant variants with semiquantitative analysis of signals intensity, normalized to level signal of housekeeping gene - GAPDH. The bars represent a mean ± SD expression of proteins. *: *p* < 0.05; **: *p* < 0.01; ***: *p* < 0.001; **** - p by Welch's *t*-test. WT- Wild Type, CDDP - cisplatin-resistant variant, NS - non-significant

To identify the involvement of small HSPs (HSPB5, HSPB6, and HSPB8) in OvCa during the development of chemoresistance, we used OvCa cell lines: A2780 and SKOV3, which represents distinct types of ovarian cancer cell lines. A2780 cell line represents the endometroid subtype with non-applied treatment. On the other hand, the SKOV3 cell line is derived from cells of a patient diagnosed with high-grade serous ovarian cancer with intrinsic resistance mechanisms to treatment. For over six months, we generated the cisplatin-resistant variants cell lines (named as follows: A2780 CDDP and SKOV3 CDDP), which were already described and characterized in a previous study (Michalak et al., 2020[37]). Briefly, we adapted cells to the concentration of cisplatin starting from 200 ng/ml to 1 µg/ml of cell culture medium. The parental cell line was maintained during the whole process of generation of CDDP-resistant cell line to exclude the genetic drift of cultured cell lines themselves. Further, among these variants, we assessed the expression of small HSPs on the transcriptome level using RT-qPCR analysis (Figure 1a and 1b(Fig. 1)). In the A2780 cell line, we observed a decreased expression of *HSPB5* in CDDP resistant variants compared to WT (Figure 1a(Fig. 1)). In the case of *HSPB6, *we did not observe significant changes between the sensitive and resistant variant, but the lowering trend in the A2780 CDDP variant was notified (Figure 1a(Fig. 1)). In the A2780 cell line, the expression of *HSPB8* was maintained at a similar level despite the induction of chemoresistance (Figure 1a(Fig. 1)). The analysis of small HSPs in the SKOV3 cell line revealed that their transcriptomic profile is different in comparison with the A2780 cell line (Figure 1b(Fig. 1)). The *HSPB5* was significantly increased in resistant variant in comparison with SKOV3 WT (Figure 1b(Fig. 1)). The analysis of *HSPB6 *expression indicated a lack of differences between SKOV3 WT and SKOV3 CDDP cell line variants (Figure 1b(Fig. 1)). On the other hand, the transcript level of *HSPB8* was higher in resistant variant than sensitive ones (Figure 1b(Fig. 1)). 

Further, to validate observed transcriptomic changes of the expression of small HSPs between sensitive and resistant to cisplatin OvCa cell lines we performed a Western blot analysis (Figure 1c and 1d(Fig. 1)). We indicated similar patterns of expression for all three proteins (HSPB5, HSPB6, and HSPB8) in A2780 CDDP compared to WT for (Figure 1c(Fig. 1)). The HSPB5 in the CDDP cells was decreased in comparison with the WT variant (Figure 1c(Fig. 1)). A similar tendency was observed in HSPB6 expression, where after induction of resistance to cisplatin, its level was downregulated in the A2780 CDDP variant compared to A2780 WT cells (Figure 1c(Fig. 1)). In the studied variants of the A2780 cell line, the barely detected expression of HSPB8 was not distinguishable between WT and induced cisplatin resistant cells (Figure 1c(Fig. 1)). In the case of small HSPs protein expression in SKOV3 cell line variants, the signal intensity of HSPB5 has slightly elevated in CDDP resistant cells (statistically non-significant) (Figure 1d(Fig. 1)). What interesting, we did observe some additional form of HSPB5 in the SKOV3 CDDP variant but not in the sensitive cells (Figure 1d(Fig. 1)). Surprisingly, we observed the downregulation of HSPB6 in the SKOV3 CDDP variant in comparison with the WT variant, which was different from its mRNA expression, where its expression was on a similar level (Figure 1d(Fig. 1)). The other small HSPs expression, HSPB8, was upregulated in the SKOV3 CDDP variant compared to WT and corresponded to the mRNA levels (Figure 1d(Fig. 1)). The small HSPs expression in cisplatin-resistant ovarian cancer cell lines differs from their sensitive variants. Moreover, the lack of similarities between trends of tested cells could be related to the distinct origin of OvCa cell lines.

### Exosomes are not the main source of small HSPs 

**Figure 2:**
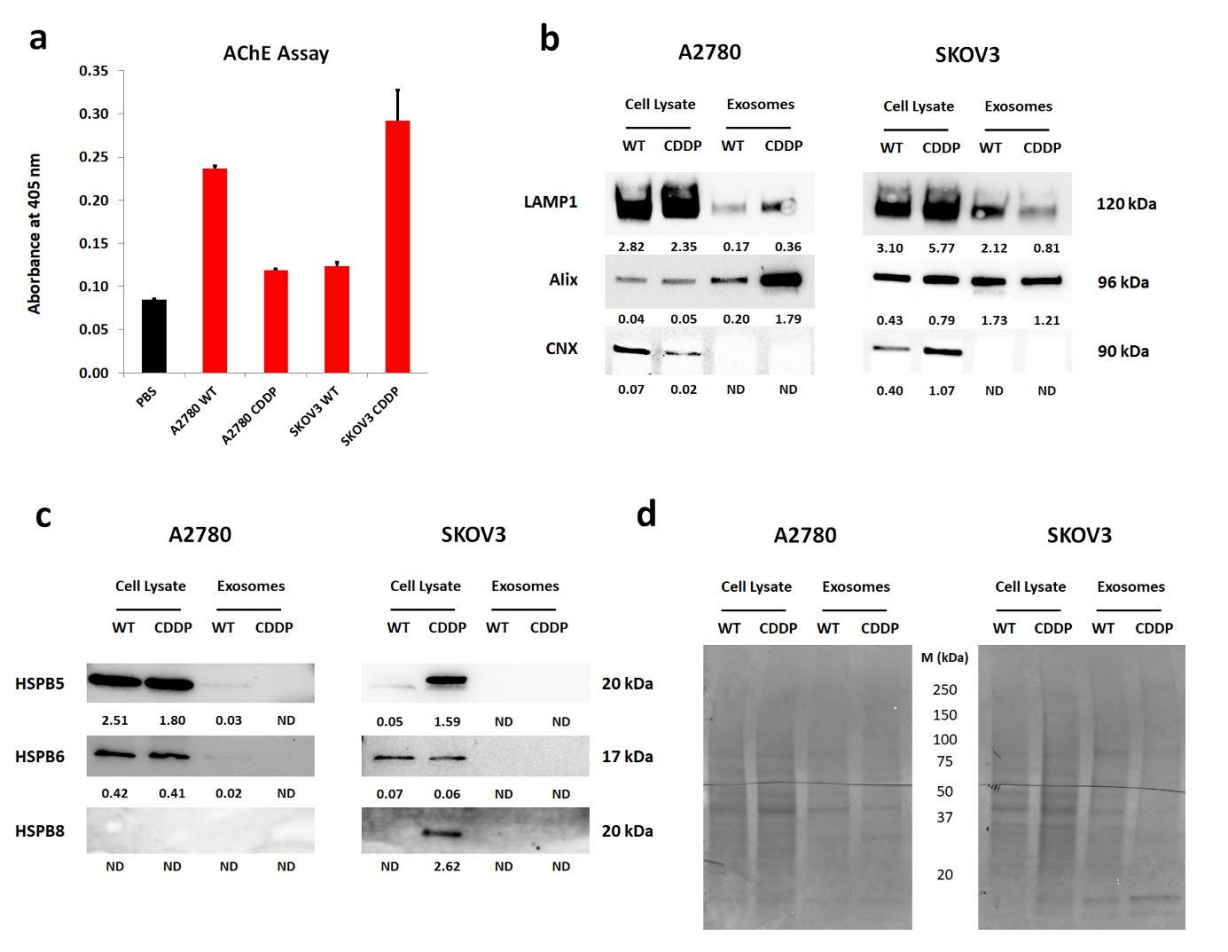
Characteristics of isolated exosomes from serum-deprived conditioned cell culture medium from sensitive and resistant to cisplatin OvCa cell lines and determination of small HSPs in their cargo. (a) The evaluation of cholinesterase activity in exosomes (5 µg) derived from cell culture of studied OvCa cell lines, PBS (diluent) was used as control. The bars represent mean ± SD from duplicate; (b) The representative Western blots describing phenotype of isolated exosomes from sensitive and resistant to cisplatin A2780 and SKOV3 cell lines conditioned culture medium. LAMP1 and Alix were used as positive control, which represents the endosomal origin of isolated particles and CNX served as a negative control, which is a protein specific for endoplasmic reticulum. The whole cell lysate was obtained from cells cultured in serum-free culture medium. The values below the detected bands determine the level of expression of tested proteins, which was normalized to the intensity of the total amount of protein of membranes stained with Ponceau; (c) Western blot analysis of isolated exosomes from sensitive and resistant to cisplatin A2780 and SKOV3 cell lines for HSPB5, HSPB6, and HSPB8. The whole cell lysate was obtained from cells cultured in serum-free culture medium.The values below the detected bands determine the level of expression of tested proteins, which was normalized to the intensity of the total amount of protein of membranes stained with Ponceau; (d) A representative picture of PVDF membranes stained with Ponceau, which indicated loading control of the same amount of protein. The band profile of cell lysates and exosomes is different. WT- Wild Type, CDDP - cisplatin-resistant variant, M - molecular protein marker, ND - not detected

Since the data suggest the transport of large subunits of HSP through the exosomal pathway and their significant role in tumor biology, we analyzed the expression profile of HSPB5, HSPB6, and HSPB8 in the cargo of exosomes derived from wild-type and cisplatin-resistant variants from both A2780 and SKOV3 cell lines (Figure 2b and 2c(Fig. 2)). 

Firstly, to diminish the contamination of exosomes from fetal bovine serum, we cultured cells in serum-free conditions to obtain the pure fraction of tumor derived-exosomes (TEX). To confirm their presence, the activity of acetylcholinesterase (AChE) was assessed (Figure 2a(Fig. 2)). The increased absorbance in isolated samples in comparison with diluent (PBS) was observed, which indicated the existence of exosomes. We observed decreasing trend in absorbance among the exosomes derived from A2780 CDDP cells compared to the WT variant (Figure 2a(Fig. 2)). The reversed trend was observed in SKOV3-derived exosomes, where the increased activity of AChE was observed in SKOV3 CDDP-derived exosomes in comparison with sensitive cells (Figure 2a(Fig. 2)). Further, to describe the phenotype of obtained exosomes and confirm the correctness of the isolation procedure, we have performed Western blot analysis for specific proteins: Lysosomal-associated membrane protein 1 (LAMP1) and ALG-2-interacting protein X (Alix) - markers related to the endosomal compartment from which they are secreted and served as a positive control (Figure 2b(Fig. 2)). To exclude the presence of co-isolation of other structures and organelles, the expression of endoplasmic reticulum-related Calnexin (CNX) was used and served as a negative marker (Figure 2b(Fig. 2)). So far there is a lack of universal housekeeping protein, which enables the comparison of the appropriate amount of protein between cell lysates and exosome fraction. Thus, we quantify the proteins using the mean signal intensity of the total lane profile (Figure 2d(Fig. 2)). The LAMP1 signal intensity was decreased in the exosomes derived from A2780 cell lines in comparison with whole-cell lysates. However, among isolated exosomes, its increased signal intensity from TEX derived from the A2780 CDDP variant was observed compared to vesicles obtained from A2780 WT cells (Figure 2b(Fig. 2)). The Alix expression was elevated in the exosomes derived from A2780 cell variants in comparison with cell lysates. Moreover, its protein content was increased in A2780 CDDP TEX than in those obtained from WT (Figure 2b(Fig. 2)). There was no signal for CNX detected in exosomes derived from conditioned medium, obtained from both A2780 cell line variants, confirming the purity of isolated exosomes and lack of contamination with organelles. In the case of the SKOV3 derived TEX, the LAMP1 expression was higher in cell lysates than in exosomes (Figure 2b(Fig. 2)). Among isolated vesicles, the signal intensity of LAMP1 was higher in TEX derived from SKOV3 WT than SKOV3 CDDP. The Alix content in the protein extracts exhibited higher signal intensity in isolated exosomes than in whole cell lysates obtained from both SKOV3 cell line variants (Figure 2b(Fig. 2)). The highest expression of Alix was notified in TEX derived from SKOV3 WT. The CNX expression was only observed in whole cell lysates but not in exosomal proteins isolated from SKOV3 cell studied variants (Figure 2b(Fig. 2)). In general, the characteristic of isolated exosomes from both OvCa cell lines and their sensitive and cisplatin-resistant counterparts has confirmed the presence of LAMP1, Alix, and the absence of CNX, which validate their appropriate isolation. Next, we checked the presence of HSPB5, HSPB6, and HSPB8 in the cargo of exosomes using the Western blot technique (Figure 2c(Fig. 2)). In general, expression of HSPB5 and HSPB6 was higher in serum-starved cells in both variants of A2780 cells than in exosomes (Figure 2c(Fig. 2)). Surprisingly, their presence in exosomes derived from A2780 WT was higher in comparison with exosomes derived from A2780 CDDP, but they were barely detectable. In the case of HSPB8, we did not detect its expression in whole cell lysates and as well in exosomes derived from serum-starved A2780 cells (Figure 2c(Fig. 2)). Surprisingly, the analysis of cargo of TEX derived from sensitive and resistant to cisplatin SKOV3 cell lines have shown a lack of studied small HSPs compared to whole cell extracts (Figure 2c(Fig. 2)). Those revelations suggest that small HSPs was not present in the exosomal cargo of cisplatin-resistant cell variants.

## Discussion

OvCa patients despite relatively good responses to first-line treatment have a high risk of relapse and development of chemoresistant phenotype. This led to tumor recurrence in about 75 % of patients (Bhoola and Hoskins, 2006[4]). The mechanism of developing resistance to first-line chemotherapy is a complex process, although there is a lack of data regarding the role of small heat shock proteins (Norouzi-Barough et al., 2018[40]; Steg et al., 2012[52]).

In this preliminary study, we have shown novel information about the potential involvement of small HSPs in the chemoresistance of OvCa, using *in vitro *model of two OvCa cell lines: A2780 and SKOV3 (sensitive and resistant to cisplatin), representing endometrioid and serous OvCa, respectively. To exclude the genetic drift during the prolonged culture to obtain stable resistance to cisplatin phenotype of OvCa cell lines, we used as control parallel cultured cells not exposed to CDDP (Michalak et al., 2020[37]). In the A2780 cell line, the expression of HSPB5 and HSPB6 was decreased in chemoresistant cells, while the expression of HSPB8 remained unchanged. In the SKOV3 cell line, we showed increased expression of HSPB8 and HSPB5 and decreased expression of HSPB6 in cisplatin-resistant variants. These results suggest the involvement of small Hsp in developing chemoresistant phenotypes of OvCa. The observed changes in the expression of tested small Hsp seem to be a characteristic trait of specific histological types of OvCa. Up to our knowledge and literature context, the incidence of small HSPs in chemoresistance to platinum-based drugs has not been directly studied. The existing data correspond to only advanced stages of disease and poor prognosis as discussed below.

The group of small HSPs is widely studied regarding their function in cellular processes particularly in the context of human diseases and cancer (Carra et al., 2017[8]; Bakthisaran et al., 2015[3]; Xiong et al., 2020[63]). The HSPB5 is responsible for the stabilization of the cytoskeleton and antiapoptotic response (Budnar et al., 2021[6]). A recent observation from analysis of clinicopathological data of OvCa patients (n=103) has shown that increased expression of HSPB5 alone/with the co-expression with p53 results in the poorer outcome and correlates with the tumor size, TNM staging, and decreased survival (Tan et al., 2019[57]). Moreover, the increased expression of HSPB5 was observed in the patients diagnosed with serous OvCa but not endometrioid, which correlates with our data obtained from the SKOV3 and A2780 cell lines (Tan et al., 2019[57]). Its increased expression is also correlated with a more invasive phenotype of colorectal and gastric cancer through induction of epithelial-mesenchymal transition (EMT) induced by activation of NF-κB or ERK signaling pathway (Li et al., 2017[34]; Chen et al., 2018[9]). However, we did not observe those changes in the A2780 cell line, which represents the endometrioid histological type OvCa. The anticancer drugs (such as cisplatin) as one of the apoptotic stimuli can lead to the mediation of reactive oxygen species (ROS) which subsequently lead to oxidative stress (Blaszczak et al., 2018[5]). It is possible that under these conditions we can observe elevated levels of stress-induced small HSPs as described above. According to recent studies regarding head and neck squamous cell carcinoma, HSPB5 is regulated by the ROS formation, which could be related to increased survival of cells and one of the possible mechanisms of unresponsiveness to the applied therapy (van de Schootbrugge et al., 2014[60]). 

The other member of small HSP, the HSB6/HSP20 has a tremendous role in skeletal, smooth and cardiac muscle physiology (Fan and Kranias, 2011[18]). In terms of malignant diseases, the studies are inconclusive and indicate the dualistic role of HSPB6. In our study, we observed the decreased expression of HSPB6 on the protein level, but unchanged in the transcript level. One of the explanations could be related to post transcription regulators. A few data suggest that hsa-miR-320 targets directly the Hsp20 and alleviates its functions (Ren et al., 2009[48]; He et al., 2015[20]). It was shown that overexpression of this specific miR has a tremendous role in the invasion and progression of OvCa (Wang et al., 2017[61]). Those revelations seem to support our results. Moreover, the decreased level of HSPB6 in the malignant tissues was correlated with advanced stages of OvCa (Qiao et al., 2014[46]). Interestingly, the level of anti-Hsp20 in the serum of OvCa patients was also significantly decreased in higher stages of the disease, which could show its crucial role in the progression of OvCa (Zhu et al., 2015[65]). Similarly, to our observation, the decreased expression of HSPB6 was also correlated with the progression of hepatocellular carcinoma and colorectal cancer (Nagasawa et al., 2014[39]; Ju et al., 2015[22]). The adverse situation was observed in non-small cell lung carcinoma (NSCLC), where its overexpression promoted tumor growth and was enabled by the downregulation of hsa-miR-320 in this type of cancer (Chen et al., 2014[9]; Lei et al., 2016[33]). 

Another important member of the HSP family, HSPB8, which is involved in cell division and uniquely in autophagy machinery, has also an ambiguous role in cancer biology (Cristofani et al., 2021[16]). In the context of platinum-resistance, its expression and role in this process are not well elucidated. In our study, we only observed a strong expression of HSPB8 in the SKOV3 CDDP variant. Recent studies presented by Suzuki's group suggest that its high expression in tissues was correlated with the invasive phenotype of serous OvCa (Suzuki et al., 2015[55]). Moreover, the same study showed that upregulation of HSP22 is responsible for increased invasive potential *via* transforming growth factor TGF-α in SKOV3.ip1 cells (Suzuki et al., 2015[55]). Elevated levels of HSPB8 was also correlated with poor prognosis among gastric and breast cancer (Shen et al., 2018[50]; Piccolella et al., 2017[44]). Recent data comparison of mRNA and miRNA from sensitive and resistant hypopharyngeal squamous cell carcinoma revealed that HSPB8 is strongly involved in the regulation of treatment response (Kong et al., 2020[26]). These data confirmed our finding that the higher content of small Hsp (in the study presented by Langdon (1995[31]) it was HSPB1 and in our case HSPB5 and HSPB8) was observed after chemotherapy, showing that chemotherapy can induce the overexpression of small HSPs (Lee et al., 2007[32]; Oesterreich et al., 1993[41]). No other studies reported the expression of small HSPs in OvCa and their involvement in chemoresistance. 

An additional goal of this study was to examine the presence of sHSP in exosomes derived from wild-type and cisplatin-resistant cell lines. Lately, exosomes have been deeply studied in the context of acquired chemoresistance by cancer cells, either by delivering the chemosensitivity-modifying cargo or by reducing the intracellular accumulation of chemotherapeutic drugs (Dong et al., 2020[17]). However, we have not detected any of the analyzed small Hsp in cisplatin-resistant variants. Only in the A2780 WT cell line, we were able to observe their small amount (HSPB5 and HSPB6). A few animal models have shown that secretion of small HSPs *via *exosomal cargo in pathophysiological events such as ischemic stroke or myocardial infarction has huge implications on their recovery (Liu et al., 2021[35]; Yu et al., 2019[64]). However, there is limited data about their involvement in malignant progression. The studies of the Kore' group (2014[27]) revealed that CRYAB (HSPB5) was upregulated in the exosomes after inflammation response in glioma cells potentially induced the antiapoptotic response to applied treatment (Kore and Abraham, 2014[27]). Moreover, they indicated that its regulation via exosomal cargo is highly related to the phosphorylation status of these proteins (Kore and Abraham, 2016[28]). Our previous results showed the expression of all sHSP (HSPB5, HSPB6, and HSPB8) in exosomes from the serum of the patients with OvCa (Wyciszkiewicz et al., 2019[62]). One of the possible explanations of this phenomenon is the low amount of sHSPs in exosomes, which we already observed in our previous analysis with serum samples (the expression was at picogram per mL level). Detecting such small amounts of proteins may require more sensitive detection methods, than those used in this study. Another possible explanation is the use of different types of samples (blood serum from OvCa patients vs. cell lines medium). The exosomes isolated from blood serum are enriched populations secreted not only by cancer cells but also by stromal cells like cancer-associated fibroblasts, tumour-associated immune cells, and also normal cells. 

This data discrepancy and the differences, which we obtained in our study, can only confirm how complicated and heterogenous the pathology of OvCa is. Our preliminary data and recently shown data provides insight into the possible interference of small HSPs in chemoresistance, suggesting that they may serve as a potential target to eliminate OvCa resistance platinum-based chemotherapy. We are aware that additional experiments regarding the administration of synthetic small HSPs or their elimination through siRNA or miRNA should be conducted in future studies focusing on global data analysis and *in vivo* evaluation to receive a holistic overview of the molecular mechanisms of sHSP-dependent chemoresistance in ovarian cancer. 

## Acknowledgements

This research benefited from a Greater Poland Cancer Center internal grant no.: 17/2017(160).

## Conflict of interest

The authors declare no conflict of interest.

## Figures and Tables

**Table 1:**
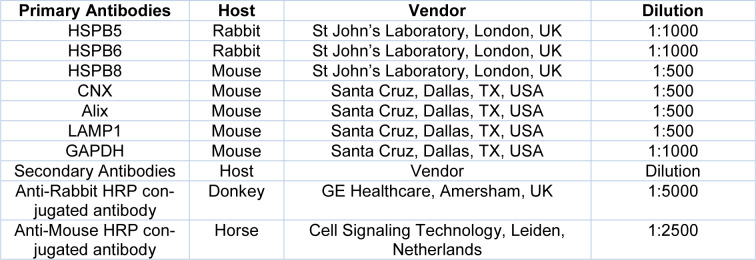
Detailed information about antibodies used in the analysis
